# Calcified plaque harboring lipidic materials associates with no-reflow phenomenon after PCI in stable CAD

**DOI:** 10.1007/s10554-023-02905-y

**Published:** 2023-06-28

**Authors:** Hayato Hosoda, Yu Kataoka, Stephen J. Nicholls, Rishi Puri, Kota Murai, Satoshi Kitahara, Kentaro Mitsui, Hiroki Sugane, Kenichiro Sawada, Takamasa Iwai, Hideo Matama, Satoshi Honda, Kensuke Takagi, Masashi Fujino, Shuichi Yoneda, Fumiyuki Otsuka, Itaru Takamisawa, Kensaku Nishihira, Yasuhide Asaumi, Kazuya Kawai, Teruo Noguchi

**Affiliations:** 1 Department of Cardiovascular Medicine, Chikamori Hospital, Kochi, India; 2 Department of Cardiovascular Medicine, National Cerebral and Cardiovascular Center, 6-1, Kishibe-Shimmachi, Suita, Osaka 564-8565 Japan; 3 MonashHeart, Monash University, Melbourne, Australia; 4 Department of Cardiovascular Medicine, Cleveland Clinic, Cleveland, OH USA; 5 Department of Cardiovascular Medicine, Sakakibara Heart Institute, Fuchyu, Tokyo Japan; 6 Department of Cardiology, Miyazaki Medical Association Hospital, Miyazaki, Japan

**Keywords:** Calcification, Lipid plaque component, No-reflow, Stable coronary artery disease, Percutaneous coronary intervention

## Abstract

Calcified atheroma has been viewed conventionally as stable lesion which less likely increases no-reflow phenomenon. Given that lipidic materials triggers the formation of calcification, lipidic materials could exist within calcified lesion, which may cause no-reflow phenomenon after PCI. The REASSURE-NIRS registry (NCT04864171) employed near-infrared spectroscopy and intravascular ultrasound imaging to evaluate maximum 4-mm lipid-core burden index (maxLCBI_4mm_) at target lesions containing small (maximum calcification arc < 180°: n = 272) and large calcification (maximum calcification arc ≥ 180°: n = 189) in stable CAD patients. The associations of maxLCBI_4mm_ with corrected TIMI frame count (CTFC) and no-reflow phenomenon after PCI were analyzed in patients with target lesions containing small and large calcification, respectively. No-reflow phenomenon occurred in 8.0% of study population. Receiver-operating characteristics curve analyses revealed that optimal cut-off values of maxLCBI_4mm_ for predicting no-reflow phenomenon were 585 at small calcification (AUC = 0.72, p < 0.001) and 679 at large calcification (AUC = 0.76, p = 0.001). Target lesions containing small calcification with maxLCBI_4mm_ ≥ 585 more likely exhibited a greater CTFC (p < 0.001). In those with large calcification, 55.6% of them had maxLCBI_4mm_ ≥ 400 [vs. 56.2% (small calcification), p = 0.82]. Furthermore, a higher CTFC (p < 0.001) was observed in association with maxLCBI_4mm_ ≥ 679 at large calcification. On multivariable analysis, maxLCBI_4mm_ at large calcification still independently predicted no-reflow phenomenon (OR = 1.60, 95%CI = 1.32–1.94, p < 0.001). MaxLCBI_4mm_ at target lesions exhibiting large calcification elevated a risk of no-reflow phenomenon after PCI. Calcified plaque containing lipidic materials is not necessarily stable lesion, but could be active and high-risk one causing no-reflow phenomenon.

**Supplementary Information:**

The online version contains supplementary material available at 10.1007/s10554-023-02905-y.

## Introduction

Lipidic plaque component is an important feature of coronary atheroma which elevates a risk of no-reflow phenomenon after PCI with stent implantation [1–3]. By contrast, calcified atheroma has been viewed conventionally as stable lesion which less likely causes this PCI-related complication. However, pathophysiologically, the formation of calcification is triggered by lipidic plaque components which induce osteogenic differentiation and inflammatory cytokines [4–9]. These features suggest that lipidic plaque materials could exist within calcified lesions, which may associate with deterioration of coronary flow after PCI. A combination catheter of intravascular ultrasound (IVUS) and near-infrared spectroscopy imaging (NIRS) is an intravascular imaging modality which enables to quantitatively visualize lipidic burden within calcified atheroma in vivo [10–14]. Therefore, the current study investigated no-reflow phenomenon after stent implantation at calcified atheroma containing lipidic plaque materials in stable CAD patients by using NIRS/IVUS imaging.

## Methods

### REASSURE-NIRS registry

**Fig. 1:**
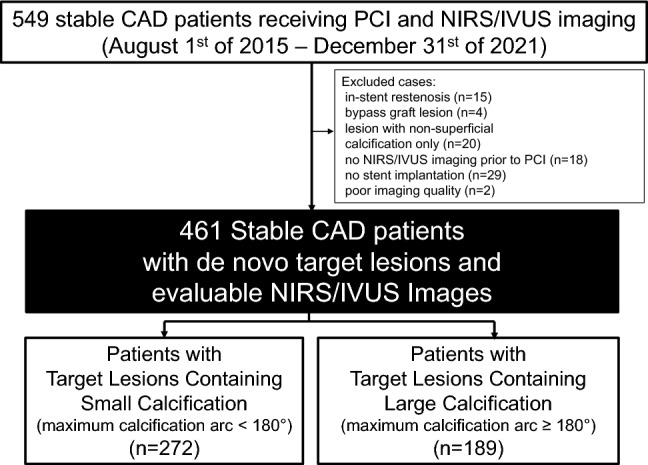
Patients’ disposition. The current study included stable CAD patients with de novo target lesions and evaluable NIRS/IVUS images. Patients were stratified into two groups according to maximum calcification arc < (small calcification) and ≥ 180° (large calcification). *CAD* coronary artery disease, *IVUS* intravascular ultrasound, *NIRS* near-infrared spectroscopy, *PCI* percutaneous coronary intervention

The REASSURE-NIRS registry is a multi-center prospective registry which consecutively enrolled patients with CAD who received PCI with NIRS/IVUS imaging (NCT04864171). A total of 529 patients with stable CAD have been enrolled in this registry from 1st of August, 2015 to 31st of December, 2021. Of these, the following subjects were excluded; subjects with in-stent restenosis at target lesions (n = 15), those with target lesion at bypass graft (n = 4), those who had lesions with non-superficial calcification only (n = 20), those who did not receive pre NIRS/IVUS imaging due to the need for pre-dilatation or debulking procedures first (n = 18), those who did not receive stent implantation (n = 29) and those with insufficient image quality (n = 2). As a consequence, the remaining 461 stable CAD patients with de novo target lesions who received PCI with drug-eluting stent (DES) implantation and NIRS/IVUS imaging were included into the current analysis (Fig. 1). Target lesion was angiographically identified. Of these, target lesions exhibiting moderate stenosis were evaluated by FFR for indication of PCI. Fractional flow reserve < 0.80 was defined as the presence of myocardial ischemia requiring PCI. Stable CAD included angina pectoris and silent myocardial ischemia. Silent myocardial ischemia was defined as fractional flow reserve ≤ 0.80. This study was approved by the institutional review board of the National Cerebral and Cardiovascular Center (M30-084-4), the Miyazaki Medical Association Hospital (2020-43) and Sakakibara Heart Institute.

### Coronary angiographic analysis and definition of no-reflow phenomenon

Coronary angiography was performed with a frame rate of 30/s. Corrected Thrombolysis In Myocardium Infarction frame count (CTFC) was measured immediately after stent implantation [15, 16]. In patients with thrombolysis in myocardium infarction flow grade 0 or 1 after PCI, a CTFC of 100 was used. No-reflow phenomenon was defined as CTFC > 27 on coronary angiography without mechanical obstruction of coronary artery after stent implantation [15, 16]. In patients who received filter-wire (Parachute™, GoodCare, Nagoya, Japan) prior to stent implantation, the occurrence of filter no-reflow was considered as no-reflow phenomenon. Coronary angiography was evaluated by two independent physicians (YK and KK). Quantitative coronary angiography (QCA) analysis was performed at target lesions by using off-line commercially available software (QAngio® XA, Medis, Leiden, the Netherlands). QCA analysis included reference vessel diameter, minimal lumen diameter, percent diameter stenosis and lesion length.

### PCI procedures and NISR/IVUS imaging

In the current study, target lesion was defined as the lesion where PCI was performed. All procedural decisions including the selection of DES, filter-wire and other devices, the use of mechanical support system and adjunctive pharmacotherapy were made according to the discretion of the individual PCI operator. Each PCI operator was encouraged to optimize stent expansion at target lesion, which was defined as (1) minimum stent area (MSA) > 5.0 mm^2^ or larger than distal reference lumen area on IVUS imaging, (2) IVUS-derived plaque area at the proximal and distal edge of the stents < 50%, and (3) no edge dissection which involves media with a length > 3 mm on IVUS imaging [17].

NIRS/IVUS imaging was conducted prior to PCI to analyze lipidic and calcific features at target lesions. In detail, after intracoronary administration of nitroglycerin (100-300ug), the imaging catheter (TVC Insight™ or Dualpro™, Infraredx, Bedford, MA, USA) was advanced into the target vessel and the catheter positioned distal to an angiographically identifiable target lesions. NIRS/IVUS imaging catheter was automatically withdrawn at a translation velocity of 0.5 mm/sec and 960 rpm (TVC Insight™) or 2.0 mm/sec and 1800 rpm (Dualpro™).

### Quantitative analysis of calcification and lipidic plaque components at target lesions on NIRS/IVUS imaging

The raw IVUS data was transferred to commercially available software, QIvus® (Medis, Leiden, the Netherlands) for quantitative analysis of IVUS images. Makoto® system (Infraredx, Bedford, MA, USA) was used to analyze obtained chemogram data on NIRS imaging. Both analyses were conducted by persons who were blinded to the clinical characteristics of the patients (HH, TI, KM, SK, KK and YK). Quantitative measurements were conducted to evaluate the degree of lipidic and calcific plaque materials at target lesions.

#### Calcification

Calcification was identified by an echogenic signal brighter than the adventitia with corresponding acoustic shadowing [18]. The arc of calcification was measured at every-1 mm cross-sectional image at target lesions. Small and large calcifications were defined as maximum calcification arc at target lesions < and ≥ 180°, respectively.

#### Lipidic plaque component

Lipidic plaque component was measured by using NIRS images. Throughout obtained raw spectra, a probability of lipid core on NIRS imaging was automatically mapped to a red-to-yellow color scale. Then, maximum 4-mm lipid-core burden index (maxLCBI_4mm_) was calculated as the number of yellow pixels within target lesions, divided by the total pixel quantity within the corresponding segments [10–13].

### Statical analysis

Continuous values with normal distribution were expressed as mean ± SD. Normality of distribution was tested with the Shapiro–Wilk test. Variables with non-normal distribution were expressed with median (interquartile range). Categorical data were expressed with n (%). Comparisons of continuous variables with normal distribution were tested by 1-way ANOVA with Tukey's multiple comparison, and variables with non-normal distribution were tested by Kruskal–Wallis test with Dunn’s nonparametric comparison with Bonferroni adjustment. The predictive ability of maxLCBI_4mm_ for no-reflow phenomenon was analyzed by receiver-operating characteristic analyses with calculations of sensitivity and specificity. The best cut-off value of maxLCBI_4mm_ was determined by selecting the value which maximized the sum of sensitivity and specificity. Spearman correlation coefficient test was used to examine relationship between maxLCBI_4mm_ and no-reflow phenomenon in patients with target lesions containing small and large calcification, respectively. Uni- and multivariable logistic analyses were performed to identify predictors for no-reflow phenomenon after PCI. All reported P values are 2-sided. P values < 0.05 was considered statistically significant. All statistical analyses will be performed using SPSS software version 27 (IBM®, Chicago, IL, USA).

## Results

**Fig. 2:**
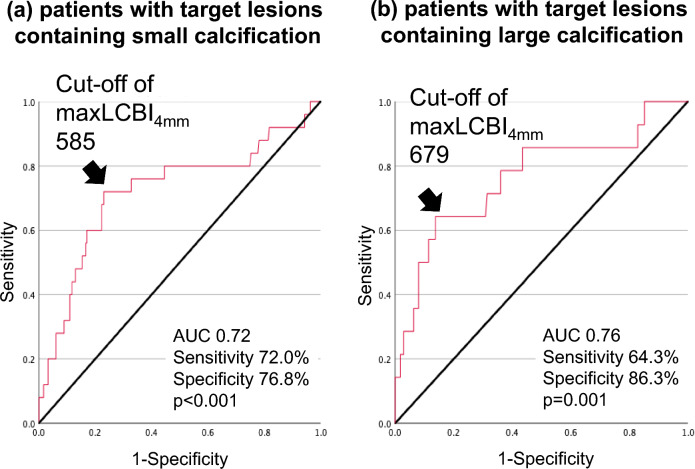
ROC analysis of MaxLCBI_4mm_ for predicting no-reflow phenomenon after PCI. The best cut-off value of maxLCBI_4mm_ to predict no-reflow phenomenon after PCI was analyzed. **a** Patients with Target Lesions Containing Small Calcification. **b** Patients with Target Lesions Containing Large Calcification. *AUC* area under the curve, *MaxLCBI*_*4mm*_ maximum 4-mm lipid-core burden index, *PCI* percutaneous coronary intervention, *ROC* receiver-operating characteristics

The current analysis included 272 and 189 stable CAD patients with target lesions containing small and large calcification, respectively (Supplementary Table). The averaged maxLCBI_4mm_ was 435 (294, 613) (p = 0.95). Of note, over 50% of subjects with large calcification had maxLCBI_4mm_ ≥ 400, which was similar to that at target lesions with small calcification (55.6 vs. 56.2%, p = 0.82). No-reflow phenomenon after PCI was observed in 8.0% of study subjects (small calcification: 9.9%, large calcification: 6.9%, p = 0.30). Table 1 presented comparison of clinical characteristics in those with and without no-reflow phenomenon after PCI. Those with no-reflow phenomenon after PCI were more likely to have a history of myocardial infarction (p = 0.02), accompanied by a lower frequency of hypertension (p < 0.001) (Table 1). On NIRS/IVUS imaging analysis, while maximum calcification arc did not differ between two groups (p = 0.68), a larger maxLCBI4mm was observed in patients who experienced no-reflow phenomenon after PCI (Table 1). In addition, the length of their implanted stents was longer with more frequent use of filter-wire (Table 1). Receiver-operating characteristics curve analyses were conducted to identify cut-off values of maxLCBI_4mm_ at target lesions which predict no-reflow phenomenon after PCI (Fig. 2). In those with target lesions containing small calcification, its value was 585 (area under the curve: 0.72, sensitivity = 72.0%, specificity = 76.8%, p < 0.001). With regard to target lesions containing large calcification, 679 was a best cut-off value (area under the curve: 0.76, sensitivity = 64.3%, specificity = 86.3%, p = 0.001) (Fig. 2).

**Table 1:** Clinical characteristics of patients with and without no-reflow phenomenon

	No-reflow phenomenon (+) (n = 39)	No-reflow phenomenon (−) (n = 422)	p value
Age (years)	67.3 ± 11.2	69.6 ± 10.8	0.23
Female, n (%)	4 (10.3)	79 (18.7)	0.27
Body mass index (kg/m^2^)	23.7 ± 3.3	24.2 ± 3.3	0.23
Hypertension, n (%)	20 (51.3)	332 (78.7)	< 0.001
Dyslipidemia, n (%)	35 (89.7)	363 (86.0)	0.63
Type 2 diabetes mellitus, n (%)	13 (33.3)	199 (47.2)	0.13
Current smoking, n (%)	9 (23.1)	79 (18.7)	0.52
Chronic kidney disease, n (%)	19 (48.7)	202 (47.9)	0.95
Hemodialysis, n (%)	2 (5.1)	26 (6.2)	0.91
A history of myocardial infarction, n (%)	17 (43.6)	108 (25.6)	0.02
A history of CABG, n (%)	2 (5.1)	21 (5.0)	1.00
Diagnosis of Stable CAD			
Angina pectoris, n (%)	12 (30.7)	122 (28.9)	0.85
Silent myocardial ischemia, n (%)	27 (69.3)	300 (71.1)	
Medication use			
Statin, n (%)	34 (87.2)	380 (90.1)	0.58
High-intensity statin, n (%)	14 (35.9)	107 (25.4)	0.18
Beta-blocker, n (%)	22 (56.4)	269 (63.7)	0.39
ACEI/ARB, n (%)	21 (53.9)	235 (55.8)	0.87
Aspirin, n (%)	35 (89.7)	400 (95.0)	0.32
P2Y12, n (%)	38 (97.4)	396 (93.8)	0.72
NIRS/IVUS-derived calcific and lipidic plaque features			
Maximum calcification arc			
0°, n (%)	9 (23.0)	68 (16.1)	0.68
0–89°, n (%)	9 (23.0)	80 (18.9)	
90–179°, n (%)	7 (18.0)	99 (23.5)	
180–269°, n (%)	7 (18.0)	99 (23.5)	
270–360°, n (%)	7 (18.0)	76 (18.0)	
Percentage of frames containing any calcification (%)	64.3 ± 41.9	67.0 ± 37.6	0.84
MaxLCBI_4mm_	649 ± 252	442 ± 220	< 0.001
PCI procedures			
Filter-wire use, n (%)	9 (23.0)	7 (1.7)	< 0.001
Rotablator, n (%)	1 (2.5)	10 (2.4)	0.98
Stent diameter (mm)	3.3 ± 0.5	3.3 ± 0.6	0.49
Stent length (mm)	37.4 ± 15.2	31.0 ± 14.9	0.006

*ACEI* angiotensin converting enzyme inhibitor, *ARB* angiotensin II receptor blocker, *CABG* coronary artery bypass grafting, *CAD* coronary artery disease, *IVUS* intravascular ultrasound, *MaxLCBI*_*4mm*_ maximum-4 mm lipid-core burden index, *NIRS* near-infrared spectroscopy imaging, *PCI* percutaneous coronary intervention

### Clinical characteristics in patients with target lesions containing small calcification

Table 2 summarizes clinical characteristics of patients with target lesions containing small calcification stratified according to a cut-off value of maxLCBI_4mm_ (585). There were no significant differences in coronary risk factors between two groups (Table 2). The frequency of guideline-recommended medications including statin was similar across the groups (Table 1).

**Table 2:** Clinical characteristics of patients with target lesions containing small calcification stratified according to MaxLCBI_4mm_ < and ≥ 585

	MaxLCBI < 585 (n = 196)	MaxLCBI ≥ 585 (n = 76)	p value
Age (years)	68 ± 10.8	68.5 ± 12.3	0.77
Female, n (%)	30 (15.3)	16 (21.1)	0.28
Body mass index (kg/m^2^)	24.3 ± 2.9	23.9 ± 3.4	0.14
Hypertension, n (%)	146 (74.5)	54 (71.1)	0.65
Dyslipidemia, n (%)	167 (85.2)	68 (89.5)	0.43
Type 2 Diabetes Mellitus, n (%)	88 (44.9)	37 (48.7)	0.59
Current Smoking, n (%)	39 (19.9)	18 (23.7)	0.51
Chronic kidney disease, n (%)	80 (40.8)	38 (50.0)	0.18
Hemodialysis, n (%)	4 (2.0)	3 (4.0)	0.40
A history of myocardial infarction, n (%)	47 (24.0)	23 (30.3)	0.28
A history of CABG, n (%)	10 (5.1)	1 (1.3)	0.30
Diagnosis of Stable CAD			
Angina pectoris, n (%)	56 (28.6)	23 (30.3)	0.78
Silent myocardial ischemia, n (%)	140 (71.4)	53 (69.7)	
Medication Use			
Statin, n (%)	176 (89.8)	70 (92.1)	0.65
High-intensity statin, n (%)	43 (21.9)	21 (27.6)	0.34
Beta-blocker, n (%)	131 (66.8)	46 (60.5)	0.33
ACEI/ARB, n (%)	106 (54.1)	47 (61.8)	0.28
Aspirin, n (%)	186 (94.9)	72 (94.7)	0.80
P2Y12 inhibitor, n (%)	186 (94.9)	72 (94.7)	0.99

*ACEI* angiotensin converting enzyme inhibitor, *ARB* angiotensin II receptor blocker, *CABG* coronary artery bypass grafting, *CAD* coronary artery disease, *MaxLCBI*_*4mm*_ maximum-4 mm lipid-core burden index

### Features of target lesions containing small calcification, and no-reflow phenomenon after PCI

**Fig. 3:**
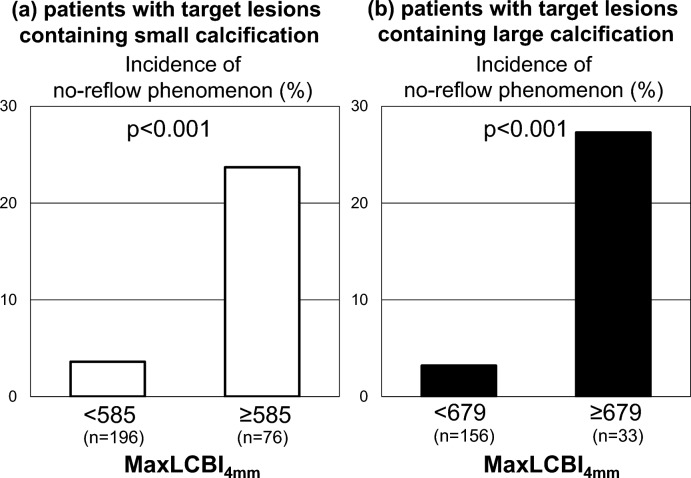
Incidence of no-reflow phenomenon. Incidence of no-reflow phenomenon after PCI was compared in subjects stratified according to maxLCBI_4mm_ at target lesions. **a** Patients with Target Lesions Containing Small Calcification. **b** Patients with Target Lesions Containing Large Calcification. *MaxLCBI*_*4mm*_ maximum 4-mm lipid-core burden index

**Fig. 4:**
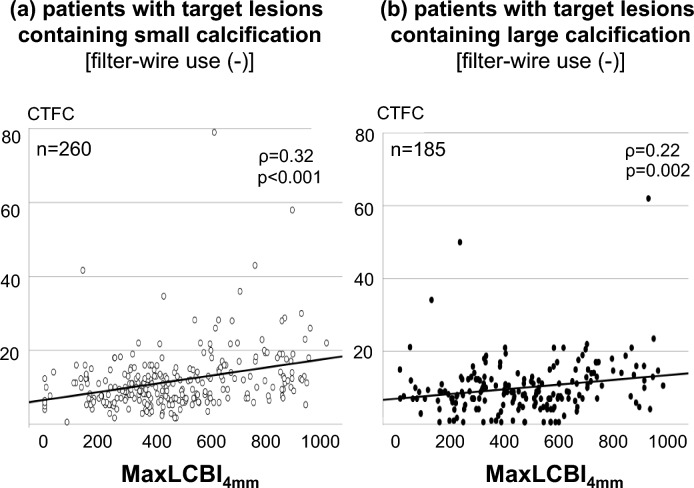
Relationship of MaxLCBI_4mm_ with CTFC in patients without filter-wire use. Spearman correlation analysis was performed to investigate the relationship between maxLCBI_4mm_ and CTFC in patients receiving PCI without filter-wire use. **a** Patients with Target Lesions Containing Small Calcification (n = 260). **b** Patients with Target Lesions Containing Large Calcification (n = 185). *CTFC* corrected thrombolysis in myocardium infarction frame count, *MaxLCBI*_*4mm*_ maximum 4-mm lipid-core burden index

Angiographic and NIRS/IVUS-derived features of target lesions containing small calcification are shown in Table 3. Over half of target lesions were located at left anterior descending artery (p = 0.87). Target lesions with small calcification exhibiting maxLCBI_4mm_ ≥ 585 were more likely to have a longer lesion length (p = 0.02), whereas maximum calcification arc did not differ in 2 groups (p = 0.77). With regard to PCI procedures, they received a longer stent length (p = 0.009) with a greater frequency of filter-wire use (p = 0.04). Following the completion of PCI, minimum lesion diameter (p = 0.54), percent diameter stenosis (p = 0.43) and IVUS-derived measures of stent expansion (minimum stent area: p = 0.41, stent expansion rate: p = 0.40) were similar between two groups (Table 3). However, subjects with maxLCBI_4mm_ ≥ 585 more likely exhibited a greater CTFC after PCI (p < 0.001, Table 3). Furthermore, as shown in Fig. 3, a risk of no-reflow phenomenon after PCI increased in associated with maxLCBI_4mm_ (23.7% vs. 3.6%, p < 0.001, Fig. 3-a). Figure 4 illustrates the relationship of maxLCBI_4mm_ with corrected TIMI frame count in patients who did not use filter-wire (n = 260). MaxLCBI_4mm_ at target lesions containing small calcification were associated with CTFC (ρ = 0.32, p < 0.001, Fig. 4a).

**Table 3:** Angiographic and NIRS/IVUS characteristics, and PCI procedures in patients with target lesions containing small calcification stratified according to MaxLCBI_4mm_ < and ≥ 585

	MaxLCBI < 585 (n = 196)	MaxLCBI ≥ 585 (n = 76)	p value
Location of culprit lesions			
LAD, n (%)	114 (58.2)	45 (59.2)	0.87
LCX, n (%)	28 (14.3)	13 (17.1)	
RCA, n (%)	52 (26.5)	52 (23.7)	
LMT, n (%)	1 (0.5)	0 (0.0)	
SVG, n (%)	1 (0.5)	0 (0.0)	
Pre PCI measures			
Quantitative coronary angiography analysis			
Reference diameter (mm)	3.0 ± 0.6	2.9 ± 0.7	0.31
MLD (mm)	0.8 ± 0.5	0.7 ± 0.4	0.47
%DS (%)	71.0 ± 16.6	72.4 ± 16.5	0.48
Lesion length (mm)	18.4 ± 10.7	23.8 ± 14.8	0.02
NIRS/IVUS-derived calcific and lipidic plaque features			
Maximum calcification arc			
0°, n (%)	54 (27.6)	23 (30.3)	0.77
0–89°, n (%)	63 (32.1)	26 (34.2)	
90–179°, n (%)	79 (40.3)	27 (35.5)	
Percentage of frames containing any calcification (%)	51.5 ± 37.9	47.6 ± 36.9	0.55
MaxLCBI_4mm_	347 ± 143	766 ± 111	< 0.001
PCI Procedures			
Filter-wire use, n (%)	5 (2.6)	7 (9.2)	0.04
Rotablator, n (%)	4 (2.1)	0 (0)	0.58
Stent diameter (mm)	3.2 ± 0.5	3.2 ± 0.6	0.78
Stent length (mm)	29.5 ± 14.4	34.8 ± 16.7	0.009
Post-dilatation after stent implantation (%)	160 (81.6)	64 (84.2)	0.71
Diameter of balloon for post-dilatation (mm)	3.4 ± 0.4	3.3 ± 0.4	0.69
Post PCI measures			
Quantitative coronary angiography analysis			
MLD after PCI (mm)	2.8 ± 0.5	2.8 ± 0.6	0.54
%DS after PCI (%)	10.0 ± 7.8	8.9 ± 7.3	0.43
Corrected TIMI frame count	9.8 ± 5.3	17.0 ± 11.4	< 0.001
The degree of stent expansion on IVUS imaging			
MSA after PCI (mm^2^)	6.5 ± 2.5	6.1 ± 2.0	0.41
Stent expansion (%)	83.6 ± 18.6	81.8 ± 15.4	0.40

*IVUS* intravascular ultrasound, *LAD* left anterior descending artery, *LCX* left circumflex artery, *MaxLCBI*_*4mm*_ maximum-4 mm lipid-core burden index, *MLD* minimum lesion diameter, *MSA* minimum stent area, *NIRS* near-infrared spectroscopy imaging, *PCI* percutaneous coronary intervention, *%DS* percent diameter stenosis, *RCA* right coronary artery, *SVG* saphenous vein graft

### Clinical characteristics in patients with target lesions containing large calcification

Table 4 describes clinical characteristics of patients with target lesions containing large calcification stratified according to maxLCBI_4mm_ (679). Similar to those with small calcifcation, the prevalence of concomitant risk factors, angina pectoris and silent myocardial ischemia, and guideline-recommended medication use was similar in 2 groups (Table 4).

**Table 4:** Clinical characteristics of patients with target lesions containing large calcification stratified according to MaxLCBI_4mm_ < and ≥ 679

	maxLCBI < 679 (n = 156)	maxLCBI ≥ 679 (n = 33)	p value
Age (years)	70.6 ± 10.3	72.9 ± 9.3	0.26
Female, n (%)	33 (21.2)	4 (12.1)	0.33
Body mass index (kg/m^2^)	23.9 ± 3.8	24.4 ± 3.2	0.38
Hypertension, n (%)	126 (80.8)	26 (78.8)	0.81
Dyslipidemia, n (%)	135 (86.5)	28 (84.9)	0.78
Type 2 Diabetes Mellitus, n (%)	74 (47.4)	13 (39.4)	0.45
Current Smoking, n (%)	27 (17.3)	4 (12.1)	0.61
Chronic kidney disease, n (%)	87 (55.8)	16 (48.5)	0.45
Hemodialysis, n (%)	18 (11.5)	3 (9.1)	0.90
A history of myocardial infarction, n (%)	43 (27.6)	12 (36.4)	0.40
A history of CABG, n (%)	10 (6.4)	2 (6.1)	0.98
Diagnosis of Stable CAD			
Angina pectoris, n (%)	45 (28.8)	10 (30.4)	0.77
Silent myocardial ischemia, n (%)	111 (71.2)	23 (69.6)	
Medication Use			
Statin, n (%)	137 (87.8)	31 (93.9)	0.54
High-intensity statin, n (%)	49 (31.4)	8 (24.2)	0.53
Beta-blocker, n (%)	94 (60.3)	20 (60.6)	0.98
ACEI/ARB, n (%)	86 (55.5)	17 (51.5)	0.70
Aspirin, n (%)	147 (94.8)	30 (90.9)	0.41
P2Y12, n (%)	144 (92.3)	32 (97.0)	0.47

*ACEI* angiotensin converting enzyme inhibitor, *ARB* angiotensin II receptor blocker, *CABG* coronary artery bypass grafting, *CAD* coronary artery disease, *MaxLCBI*_*4mm*_ maximum-4 mm lipid-core burden index

### Features of target lesions containing large calcification, and no-reflow phenomenon after PCI

In patients with target lesions containing large calcification, the location of target lesions, quantitative coronary angiographic measures and the proportion of maximum calcification arc did not differ in two groups (Table 5). Filter-wire was more frequently used in those with maxLCBI_4mm_ ≥ 679 (p = 0.01). On angiographic analysis after PCI, those with maxLCBI_4mm_ ≥ 679 more likely exhibited larger minimum lumen diameter (p = 0.02) and corrected TIMI frame count (p < 0.001). Furthermore, large calcification with maxLCBI_4mm_ ≥ 679 increased a risk of no-reflow phenomenon (27.3% vs. 3.2%, p < 0.001, Fig. 3b). A significant relationship between maxLCBI_4mm_ at target lesions containing large calcification and CTFC existed (ρ = 0.22, p = 0.002) in subjects who did not use filter-wire (n = 185) (Fig. 4b).

**Table 5:** Angiographic and NIRS/IVUS characteristics, and PCI procedures in patients with target lesions containing large calcification stratified according to MaxLCBI_4mm_ < and ≥ 679

	maxLCBI < 679 (n = 156)	maxLCBI ≥ 679 (n = 33)	p value
Location of culprit lesions			
LAD, n (%)	100 (64.1)	17 (51.5)	0.62
LCX, n (%)	18 (11.5)	6 (18.2)	
RCA, n (%)	35 (22.4)	1 (3.0)	
LMT, n (%)	2 (1.3)	0 (0.0)	
SVG, n (%)	1 (0.7)	9 (27.3)	
Pre PCI measures			
Quantitative coronary angiography analysis			
Reference diameter (mm)	2.9 ± 0.7	3.2 ± 0.8	0.10
MLD (mm)	0.8 ± 0.4	0.71 ± 0.4	0.18
%DS (%)	70.9 ± 14.2	76.0 ± 13.0	0.10
Lesion length (mm)	19.3 ± 12.7	23.8 ± 15.6	0.17
NIRS/IVUS-derived calcific and lipidic plaque features			
Maximum calcification arc			
180–269°, n (%)	89 (57.1)	17 (51.5)	0.57
270–360°, n (%)	67 (42.9)	16 (48.5)	
Percentage of frames containing any calcification (%)	96.0 ± 11.7	97.6 ± 8.8	0.50
MaxLCBI_4mm_	379 ± 172	800 ± 93	< 0.001
PCI procedures			
Filter-wire use, n (%)	1 (0.6)	3 (9.1)	0.01
Rotablator, n (%)	5 (3.2)	2 (6.1)	0.35
Stent diameter (mm)	3.3 ± 0.6	3.5 ± 0.6	0.56
Stent length (mm)	31.6 ± 14.1	36.0 ± 16.6	0.24
Post-dilatation after stent implantation (%)	148 (94.8)	31 (93.9)	0.90
Diameter of balloon for post-dilatation (mm)	3.4 ± 0.5	3.6 ± 0.6	0.69
Post PCI measures			
Quantitative coronary angiography analysis			
MLD after PCI (mm)	2.7 ± 0.6	3.0 ± 0.5	0.02
%DS after PCI (%)	11.6 ± 11.2	9.8 ± 7.1	0.68
Corrected TIMI frame count	9.0 ± 6.3	14.9 ± 9.6	< 0.001
The degree of stent expansion on IVUS imaging			
MSA after PCI (mm^2^)	6.6 ± 2.2	6.2 ± 2.3	0.37
Stent expansion (%)	79.2 ± 20.5	78.1 ± 14.0	0.63

*IVUS* intravascular ultrasound, *LAD* left anterior descending artery, *LCX* left circumflex artery, *MaxLCBI*_*4mm*_ maximum-4 mm lipid-core burden index, *MLD* minimum lesion diameter, *MSA* minimum stent area, *NIRS* near-infrared spectroscopy imaging, *PCI* percutaneous coronary intervention, *%DS* percent diameter stenosis, *RCA* right coronary artery, *SVG* saphenous vein graft

### Predictors of no-reflow phenomenon after PCI

**Fig. 5:**
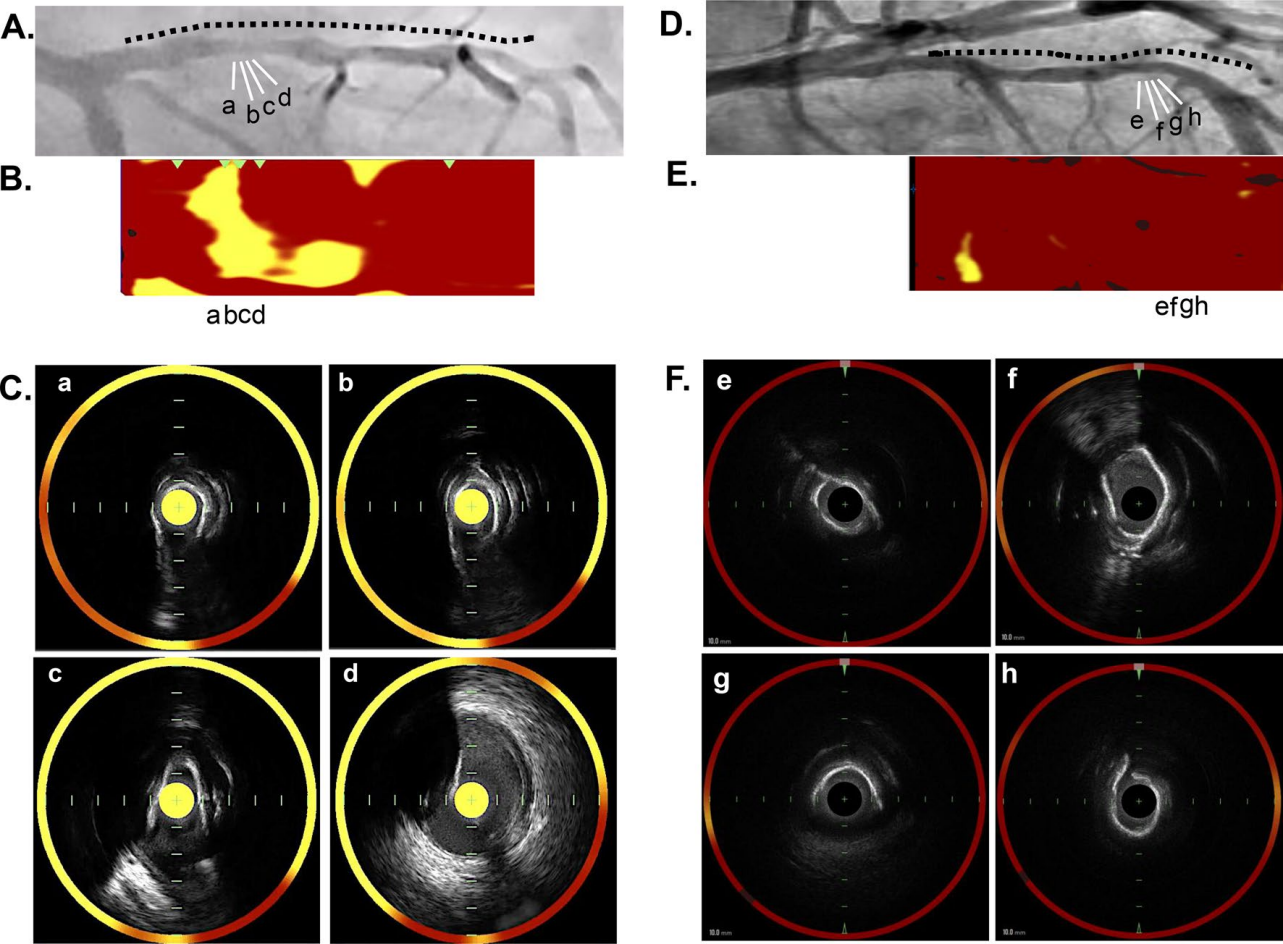
Representative cases. **A** A 67-year old gentleman presented stable angina pectoris. The proximal segment of his LAD was treated by PCI. Black dotted line was the imaged segments with NIRS/IVUS. a, b, c and d correspond to images in **B** and **C**. **B** NIRS imaging prior to PCI visualized the presence of lipidic plaque materials at the calcified target lesion, and maxLCBI_4mm_ was 817. **C** Cross-sectional images (**D**) showed the extensive yellow signals at the corresponding calcified lesion. The maximum calcification arc was 297°. After stent implantation, no-reflow phenomenon occurred. **D** A 58-yearl old gentleman received an elective PCI to treat moderate stenosis at the middle segment of his LAD. Black dotted line was the imaged segments with NIRS/IVUS. e, f, g and h correspond to images in **E** and **F**. **E** MaxLCBI_4mm_ at this lesion was 83. **F** On NIRS/IVUS cross-sectional imaging, calcification arc was 360° (**H**), accompanied by very small amount of yellow signals. No-reflow phenomenon did not occur after PCI. *IVUS* intravascular ultrasound, *LAD* left anterior descending artery, *MaxLCBI*_*4mm*_ maximum 4-mm lipid-core burden index, *NIRS* near-infrared spectroscopy, *PCI* percutaneous coronary intervention

Uni- and multivariable logistic analyses were conducted to elucidate predictors of no-reflow phenomenon after PCI (Table 6). In patients with target lesions containing small calcification, univariate analysis showed age [odds ratio (OR) = 0.96, 95% confidence interval (CI) = 0.92–0.99, p = 0.03], a history of hypertension (OR = 0.34, 95%CI = 0.15–0.80, p = 0.01) and maxLCBI_4mm_ (OR = 1.41, 95%CI = 1.25–1.68, p < 0.001) as significant predictors (Table 6-a). Even after adjusting age, female gender, a history of hypertension, %DS after PCI and MSA, maxLCBI_4mm_ remained an independent predictor of no-reflow phenomenon (OR = 1.35, 95%CI 1.14–1.63, p = 0.007, Table 6a). In patients with target lesions containing large calcification, following adjustment of clinical characteristics (age, female gender, a history of hypertension, %DS after PCI and MSA), maxLCBI_4mm_ still continued to associate with no-reflow phenomenon (OR = 1.60, 95%CI = 1.32–1.94, p < 0.001, Table 6b). Figure 5 illustrates two representative cases.

**Table 6:** Predictors of no-reflow phenomenon

Variables	OR	95%CI	p value	Variables	OR	95%CI	p value
Patients with non-calcified target lesions							
Age	0.96	0.92–0.99	0.03	Age	0.9	0.91–0.99	0.04
Female	0.66	0.19–2.31	0.51	Female	1.213	0.24–6.12	0.81
Body mass index	1.01	0.87–1.15	0.91	–	–	–	–
Hypertension	0.34	0.15–0.80	0.01	Hypertension	0.35	0.12–1.03	0.06
Dyslipidemia	1.90	0.43–8.45	0.39	–	–	–	–
Type 2 Diabetes Mellitus	0.52	0.21–1.26	0.15	–	–	–	–
Chronic kidney disease	1.02	0.44–2.33	0.96	–	–	–	–
Statin	0.75	0.21–2.72	0.66	–	–	–	–
%DS	0.99	0.97–1.02	0.87	–	–	–	–
MLD	1.35	0.49–3.70	0.55	–	–	–	–
Lesion length	1.01	0.97–1.04	0.62	–	–	–	–
Calcium score	0.73	0.44–1.21	0.23	–	–	–	–
MaxLCBI_4mm_ per 100	1.41	1.25–1.68	< 0.001	MaxLCBI_4mm_ per 100	1.35	1.14–1.63	0.007
%DS after PCI	1.05	0.99–1.11	0.06	%DS after PCI	1.05	0.99–1.12	0.07
MLD after PCI	0.75	0.30–1.87	0.54	–	–	–	–
MSA	1.08	0.92–1.26	0.30	MSA	1.18	0.99–1.40	0.06
% stent expansion	1.01	0.98–1.03	0.35	–	–	–	–
Patients with calcified target lesions							
Age	1.02	0.97–1.08	0.36	Age	1.05	0.97–1.14	0.18
Female	0.29	0.03–2.34	0.25	Female	0.31	0.01–1.34	0.55
Body mass index	0.86	0.72–1.04	0.13	–	–	–	–
Hypertension	0.20	0.06–0.63	0.006	Hypertension	0.08	0.01–0.43	0.003
Dyslipidemia	0.95	0.20–4.52	0.95	–	–	–	–
Type 2 Diabetes Mellitus	0.63	0.20–1.95	0.42	–	–	–	–
Chronic kidney disease	1.12	0.37–3.37	0.83	–	–	–	–
Statin	0.73	0.15–3.51	0.69	–	–	–	–
%DS	1.02	0.98–1.06	0.27	–	–	–	–
MLD	0.71	0.14–3.66	0.69	–	–	–	–
Lesion length	0.99	0.95–1.04	0.92	–	–	–	–
Calcium score	1.30	0.43–3.87	0.63	–	–	–	–
MaxLCBI_4mm_ per 100	1.65	1.44–2.01	< 0.001	MaxLCBI_4mm_ per 100	1.60	1.32–1.94	< 0.001
%DS after PCI	0.98	0.92–1.04	0.59	–	–	–	–
MLD after PCI	1.31	0.49–3.48	0.58	–	–	–	–
MSA	0.87	0.65–1.16	0.35	MSA	0.89	0.63–1.26	0.52
% stent expansion	0.99	0.967–1.02	0.86	–	–	–	–

*MaxLCBI*_*4mm*_ maximum-4 mm lipid-core burden index, *MLD* minimum lesion diameter, *MSA* minimum stent area, *%DS* percent diameter stenosis, *RCA* right coronary artery

## Discussion

Calcified lesion has been characterized as quiescent atheroma with a lower risk of no-reflow phenomenon after PCI. In the current study, however, this PCI-related complication occurred even at target lesions containing large calcification in stable CAD patients. A risk of no-reflow phenomenon after PCI at those with large calcification was driven by the degree of lipidic materials. MaxLCBI_4mm_ on NIRS imaging was an independent predictor of no-reflow phenomenon even after adjustment of clinical, angiographic and IVUS features. Our findings suggest that lipidic materials could exist within calcified lesions and associate with an increased risk of no-reflow phenomenon after PCI with DES implantation.

Pathohistological studies have shown that calcification presents around inflamed lipid-rich necrotic cores [4–9], whereas in vivo studies exploring the colocalization of these plaque features were limited. The current study employed NIRS imaging which enables to evaluate lipidic materials under overlying calcification due to the ability of near-infrared light to penetrate calcification. We observed that 55.6% of calcified target lesions exhibited maxLCBI_4mm_ > 400, a signature of lipid-rich lesion, in stable CAD patients, and this frequency was similar to that at non-calcified target lesions (= 56.2%). The interactions between calcific and lipidic plaque components have been recently reported by one recent study conducting frame-based analysis. This study reported the presence of both calcium and lipids at 9.3% of analyzed frames at non-culprit segments in 139 patients with CAD selected from the IBIS-3 study [14]. The frequency of colocalized calcium and lipid varies in this and our studies partly due to different analytic method and evaluated lesions. However, these findings highlight that calcified lesion is not necessarily quiescent atheroma, but it could exhibit active form harboring lipidic material. Whether a risk of future coronary events differs in calcified lesions with and without lipidic plaque components requires further investigation.

The current study observed an increased risk of no-reflow phenomenon at lesions containing large calcification in association with the degree of lipidic materials. Furthermore, the relationship between deteriorated coronary flow and maxLCBI_4mm_ at lesions containing large calcification was identified. Mechanistically, a dynamic fracture of overlying calcification after DES implantation might occur, which allows lipidic materials within the lesions to embolize and then cause deterioration of coronary flow. It could be speculated thickness of calcification as a potential contributor to this PCI-related modification of calcified lesions. Due to a limited resolution of IVUS, the current study was not able to investigate how calcification overlying lipidic plaque components behaved PCI. Further characterization of calcification is warranted by using other imaging technique.

Intravascular imaging modalities have been shown as a tool to estimate a risk of no-reflow phenomenon after PCI [2, 19, 20]. A greater frequency of no-reflow phenomenon was observed at lesions with IVUS-derived ultrasound attenuation [16], necrotic core visualized by VH-IVUS [20] or lipid plaque on OCT imaging [2]. In the current study, maxLCBI_4mm_ at lesions with both small and large calcification predicted this PCI-related complication. MaxLCBI_4mm_ has been histologically validated to reflect the presence of lipid-rich plaque [10–14], and this NIRS-derived measure corresponds to the aforementioned plaque features visualized by IVUS, VH-IVUS or OCT [21, 22]. These collective findings support the potential of NIRS imaging to identify high-risk coronary lesions causing no-reflow phenomenon after PCI.

There are several published studies which reported cut-off values of NIRS-derived measure to predict no-reflow phenomenon. Goldstein, et al. analyzed the relationship of maxLCBI_4mm_ with periprocedural myocardial infarction in 62 CAD patients receiving PCI [23]. In this analysis, maxLCBI_4mm_ ≥ 500 was a predictor of periprocedural myocardial infarction. The another study investigated the predictive ability of a novel index, total LCBI/maxLCBI_4mm_ ratio for filter-no-reflow phenomenon in 32 ACS patients [24]. Total LCBI/maxLCBI_4mm_ ratio > 0.42 was an independent predictor of filter-no-reflow phenomenon. While these studies revealed clinical applicability of maxLCBI_4mm_ to estimate a risk of PCI-related complication risk, whether calcification affects the relationship of this NIRS-derived measure with no-reflow phenomenon has not been investigated. The current findings provide additional evidence which indicates maxLCBI_4mm_ at calcified lesions as a tool to stratify a risk of no-reflow phenomenon after PCI.

High maxLCBI_4mm_ at calcified lesions underscores adjunctive management to mitigate a risk of no-reflow phenomenon in stable CAD patients receiving PCI. We reported a case using a filter-wire at severely calcified lesion with substantially elevated maxLCBI_4mm_, which was effective to prevent coronary flow deterioration after DES [25]. More awareness is required for interventionalists to consider embolic protection device for treating calcified target lesions containing lipid-rich materials in stable CAD patients. However, this approach is not available when rotational or orbital atherectomy is used to modulate severe calcification. It may be better to consider debulking procedure which focuses on a limited segment with appropriate size of device and rotational/orbital speed. Given a greater frequency of maxLCBI_4mm_ > 400 even at target lesions containing large calcification, more intensified lipid-lowering therapies are required prior to PCI.

IVUS enables to detect calcification. However, this modality is limited to quantify calcification volume due to acoustic shadowing. Other invasive and non-invasive imaging (optical coherence tomography, computed tomography, etc.) are capable of visualizing calcification but not measuring its volume. Novel imaging approach quantifying calcification volume is required to further elucidate whether calcification volume itself affects the relationship between maxLCBI_4mm_ and no-reflow phenomenon.

## Study limitations

Several caveats should be considered to interpret the current findings. Firstly, this is a retrospective cross-sectional observational study from a registry database which enrolled stable CAD patients using NIRS/IVUS imaging for PCI, therefore selection bias could not be excluded. Secondly, procedural strategy including filter-wire was decided according to each PCI operator. This may be bias affecting PCI outcomes. Thirdly, the current study included stable CAD patients only. The cut-off values of maxLCBI_4mm_ at target lesions for predicting no-reflow phenomenon may be different in ACS patients. Fourth, IVUS does not have capability to evaluate thickness and volume of calcification. It remains unknown whether these features of calcification could affect the current findings. Fifth, the current study evaluated CTFC immediately after DES implantation but not pre-dilatation or debulking procedure. This is because coronary flow could be deteriorated due to severe dissection and/or thrombus caused by plain old balloon angioplasty and/or rotational/orbital atherectomy. Therefore, it remains unknown the frequency of no-reflow phenomenon after procedure prior to DES implantation. Sixth, the size of NIRS imaging catheter’s shaft is a larger compared to other IVUS catheter (Altaview™, Terumo, Japan) and OCT. In addition, since NIRS/IVUS catheter has a fiber cable which emits near-infrared light, this makes its catheter less flexible. These features of NIRS/IVUS imaging catheter makes difficult to cross lesions containing heavy calcification. In the current study, NIRS/IVUS was not used at lesions exhibiting very severe calcification. Therefore, it remains unknown about the frequency and characteristics of lipidic plaque materials at lesions containing very severe calcification.

## Conclusions

In the present study analyzing stable CAD patients, maxLCBI_4mm_ at lesions containing small calcification predicted the occurrence of no-reflow phenomenon after PCI. Of note, even at target lesions harboring large calcification, 55.6% of those exhibited maxLCBI_4mm_ > 400. Moreover, despite features of calcification, maxLCBI_4mm_ at target lesions with large calcification was associated with a higher CTFC and a greater frequency of no-reflow phenomenon after PCI. Our findings suggest that calcified plaque concomitantly harbors lipidic plaque materials in vivo. Calcified plaque containing lipidic materials is not necessarily stable lesion, but could be active and high-risk one causing no-reflow phenomenon.

### Supplementary Information

Below is the link to the electronic supplementary material.

Supplementary file1 (DOCX 14 KB)

## Data Availability

The data sharing underlying this article requires the approval of principal investigator and the research ethics committee at National Cerebral & Cardiovascular Center.

## Abbreviations

CAD: Coronary artery disease
CI: Confidence interval
CTFC: Corrected thrombolysis in myocardium infarction frame count
DES: Drug-eluting stent
IVUS: Intravascular ultrasound
MaxLCBI_4mm_: Maximum 4-mm lipid-core burden index
NIRS: Near-infrared spectroscopy
OR: Odds ratio
PCI: Percutaneous coronary intervention
