# Supplementary material for: Calcified plaque harboring lipidic materials associates with no-reflow phenomenon after PCI in stable CAD
Source: Int J Cardiovasc Imaging. 2023 Jun 28;39(10):1927–41. doi: 10.1007/s10554-023-02905-y (PMC10589149; doi:10.1007/s10554-023-02905-y)
Supplement: Supplementary file 1 — Supplementary file1 (DOCX 14 KB) [file 10554_2023_2905_MOESM1_ESM.docx]

**Supplementary Table. MaxLCBI_4mm_ and the Frequency of No-reflow Phenomenon in Stable CAD Patients with Non-Calcified and Calcified Target Lesions**

|  | Overall  (n=461) | Patients with  Target Lesions  Containing Small Calcification  (n=272) | Patients with  Target Lesions  Containing Large Calcification  (n=189) | p-value |
| --- | --- | --- | --- | --- |
| maxLCBI_4mm_ * | 435 (294, 613) | 431 (304, 614) | 451 (281, 600) | 0.95 |
| >400, n (%) | 258 (55.9) | 153 (56.2) | 105 (55.6) | 0.82 |
| No-reflow phenomenon, n (%) | 37 (8.0) | 25 (9.9) | 12 (6.9) | 0.30 |

* Values are medians (interquartile range)

CAD = coronary artery disease, MaxLCBI_4mm_ = maximum 4-mm lipid-core burden index
